# Report of the Discussions before the Kentucky State Dental Association

**Published:** 1880-10

**Authors:** 


### REPORT OF THE DISCUSSIONS BEFORE THE KENTUCKY STATE DENTAL ASSOCIATION, JUNE 2ND AND 3RD, 1880.

[CONTINUED FROM PAGE 369.]

### FIRST DAY—AFTERNOON SESSION.

### *Subject*—DISEASE OF THE ANTRUM.

*Dr. Taft.*—This is a subject of interest and importance to the dentist; it is so because of its frequent occurrence, because of its severity, because of its obscurity, and because it is so imperfectly understood. It has not been investigated and studied as many other affections. Physicians and dentists have too often been content to nominate it ague of the face, facial rheumatism, or neuralgia, or some other name about as far from the truth. And the treatment of course in such instances will correspond with the views entertained about it.

The antrum of Highmore, a cavity in the superior maxilla, having a capacity of from one to two fluid ounces, in the adult, is the seat of various affections.

The most simple, and the more common of its affections is simple engorgement.

This occurs by irritation of the lining membrane, together with closure of the natural channel, from the cavity into the nostril.

This irritation may occur by extension from the inflamed membrane of the nostril, or it may, and often does take place, from the perforation of the floor of the cavity by the diseased roots of teeth. These are the common sources of the simple affections of this cavity. The membrane lining the exit canal may become thickened and so close the canal and prevent the out-flow of the normal secretion. This may take place while there is little or no irritation of the lining of the antrum. But in most instances there will be an early vitiation of this retained secretion, and this will be a source of irritation to the membrane.

Disease is sometimes produced in this cavity by extension of disease from surrounding parts or tissues; for instance tumors of the gums may extend into this space and involve its membrane and walls in disease. Such extension may also occur from the alveolus or any of the adjacent hard tissues. It is important to understand the sources from which the various affections of this cavity may arise.

Simple engorgement will seldom produce pain, especially during the earlier periods. But it may cause some distension of the walls, and occasion enlargement or fullness of the cheek. Then there may be experienced a sense of fullness and uneasiness.

This only occurs where there is a freedom from a systemic cachixia, and a good degree of constitutional vigor.

The treatment of simple engorgement, especially when taken early, consists mainly in evacuation. This may be effected some times by freeing the natural channel into the cavity, and this, can in some cases, be done by reducing the inflamed and thickened condition of the mucous membrane of the nostril. In other cases perforation of the canal should be made with a curved probe. This operation requires for its right performance, skill, and a properly shaped instrument.

When these methods are impracticable entrance to the antrum should be made through the sockets of one of the molar teeth. And usually the most favorable for this is the alveolus of the posterior buccal root of the first molar. The floor of the antrum at this point is exceedingly thin. Indeed, in many cases, the point of the root perforates it. The question of the removal of a sound and healthy tooth for this purpose arises at this point: in none except extreme cases should this be done. Frequently there will be one or more teeth missing at a point through which an opening may be effected; again there may be diseased teeth or roots in the part, that may be removed. Rarely, if ever, should a sound tooth be removed for the treatment of the more, simple form of disease of the antrum. An opening may be made through the anterior wall of the cavity at its inferior edge, on a line with the space between the roots of the first molar and second bicuspid; very seldom is this operation required. If a superior molar, though sound, has lost its antagonist of the lower jaw, it would be better to extract it, to effect an entrance, than to open through the anterior wall. After an opening has been made, and good drainage obtained, simple washing with a mild soap solution, or a mild solution of common salt, may facilitate the return to a healthy condition.

This much I have said to prepare the way for further discussion of the subject.

*Dr. A. W. Smith.*—I had case of diseased antrum some time ago, of about four years' standing. Patient applied to me to be treated for neuralgia.

The first to third molars had been extracted. He complained of pain in the superior maxillary bone. After very careful examination I discovered a small fistulous opening about the size of a large-size knitting needle. I introduced a probe, and upon withdrawing it there was a free discharge, and very offensive. I at once concluded it was a case of diseased antrum. I treated it with sulphate of zinc, and like remedies. I fortunately cured the disease, the secretions dried up, and the antrum seemed in a perfectly healthy condition; but the fistula did not close up; did not know whether I had treated it right. I saw the patient about six or seven months afterwards. He said he had had no pain, but the fistula remained, and he could draw the air through it. Physician treated constitutionally. That helped him some. After about two years he caught cold and the disease returned. I concluded there must be some necrosed bone there. I cut down and made a large opening; while open felt for detached bone; found it; removed the bone, and the place healed, but the fistula remained. I would like Prof. Taft to explain the treatment.

*Dr. Taft.*—Well, if you succeeded in restoring your patient, and all satisfactory, you pursued about the right treatment, perhaps. You might have varied the applications with some advantage. In that case the canal would have closed in all probability. I think you omitted one thing in not looking for necrosed bone in the first place. It is well to look for this always. Then another thing you did not open up large enough in the first place. Was there any sphilitic taint? Was the vitality good?

*Dr. A. W. Smith.*—Vitality was very poor. The constitutional treatment helped very much. He was all run down, so much so he was not able to labor. After the operation he was able to labor. I would like to know the remedies.

*Dr. Taft.*—Well, first, wash out the cavity thoroughly. To do this you must have a large free opening. If your patient is timid and afraid of being cut, get some of the "Surgeons' Sea Tangle;" trim it down to fit the opening closely; press it up well and let it remain; in a short time it will expand and make an opening as large as you want. Wash out thoroughly, then use disinfectants. Carbolic acid is good. Salicylic acid is good. Disinfect thoroughly; use antiseptics; wash out well; prevent the vitiation of the secretion. Sometimes escharotics are called for. Chloride of zinc, nitrate of silver is employed. Iodine not much use in the early stage. When parts need stimulation then it is very well. In mild cases, use mild treatment. In severe cases, use stronger treatment. Use constitutional treatment; tone up the system; give good food; give plenty of good air and exercise. All these are of great benefit.

*Dr. Canine.*—I have had a good deal of experience with disease of the antrum, and I approve of the large free opening. I remember one case where a piece of bone had been driven through into the cavity by a blow. The case had been under the treatment of a physician for some time. I opened up through where the first molar had stood; made an opening that I could have run the large end of a plugger through; got the bone out, and all went well. I think the trouble generally comes in not opening free enough. Dr. Taft has given you the treatment.

*Dr. Dedman.*—I had a patient come into my office. His son was a physician, and I extracted a tooth for him, and the opening never closed. The cavity extended into the antrum.

*Dr. Rawls.*—That is one of the conditions in which it is very hard to suggest any mode of treatment that will secure a restoration of the parts. It is well known that the secretions of the cavity will prevent the closing up of the parts, and an opening in the lower wall was never known to be filled up by any physiological action.

*Dr. H. A. Smith.*—I would call the gentleman's attention to Dr. Farrar's tube, a full description of which is given in the Dental Cosmos. I agree with Prof. Taft, as to the large opening, but I think it a very good plan to have a tube introduced into the cavity, having it closed at the lower end so that it could be opened and the cavity cleansed without disturbing the parts. Have the tube just enter the cavity, having it attached to the teeth, so it would remain in its place. I think one mistake we make in treating diseases of the antrum is in not having a tube. If the case needed medication I would change the character of the secretions. There may be catarrhal secretions. I sometimes use salt and water. I would treat but once, and then heroically, give constitutional treatment, and then allow rest. Very frequently a patient needs nothing but rest. In Dr. Smith's case I should have put that man on his back and made him rest. He is worn down and needs rest. Very frequently harm is done by over treating.

*Dr. Canine.*—I always use the tube.

*Dr. H. A. Smith.*—It is very important not to have the tube enter the cavity too far, or the secretions will collect around it. Let it just enter the floor of the cavity so that the secretions may pass in freely. As Dr. Taft states, it is well to first find out if there is any dead bone. If there is, remove it. Don't be afraid of hurting your patient.

*Dr. Taft.*—In answer to the question by Dr. Dedman. If you will enlarge the opening and find out what is the condition of the bone, and if there is any dead bone remove it, and then apply some escharotic, you will then find the opening will close. Sometimes there will be a piece of root left, which will prevent the soft part from closing.

I have recently, instead of the tube, used hemp or flax, or lint, saturated with some antiseptic and crowded into the cavity. It serves as a vehicle for any preparation used in the treatment. As Dr. Smith says, be careful not to over-treat.

*Dr. Canine.*—Would there not be danger in general practice in using cotton in that way, by letting some portions remain in the cavity ? In the hands of some men, I mean.

*Dr. Taft.*—No, not if the material is of a coarse fiber, cotton would not answer. There is danger with some men in doing anything. There is danger with some men in filling teeth.

*Dr. Rawls.*—Mr. President, I havn't anything special to say, but I don't want the profession led astray—especially the younger members. I don't believe it is possible to close the opening in the walls of the cavity. One difficulty in the way of closing the cavity, is the secretions are passing through constantly.

*Dr. Peabody.*—Dr. Rawls is right on general principles. Some years ago I had a tooth extracted. There was no disease of the antrum. It all healed, the parts closed—that is, they came together. But for ten years I could not eat watermelon without some of it coming out at the nose. It never troubled me at any other time. Watermelon was the only thing that seemed to pass through. I thought some times if I would make a plate to impinge on the gum, I might bring the parts together, and make it close, but I have not done it.

*Dr. Taft.*—Well, Mr. President, I don't want the profession led astray, and made to believe that these openings never close. I know in some cases they do close, and close absolutely.

*Dr. A. W. Smith.*—In the case I just described the opening has closed. I don't think the theory of Dr. Rawls will hold good.

*Dr. H. A. Smith.*—I would like Dr. Rawls to explain why we can't have a closure—what prevents it.

*Dr. Rawls.*—It is the result of the flow of the secretions. I am speaking now of the lowest portion of the antrum—the floor of the antrum. We have ragged edges of the bone, some of the tissues broken, and we can't have a union of the parts, for they are not supplied with the proper nutrition that favors granulation. The continued flow of the secretions prevents granulation. And even if you have granulations those granulations are very weak and will break down. You must have pabulum thrown out. The parts are not supplied with the proper nutrition.

*Dr. Taft.*—I must enter protest to the position of Dr. Rawls. Now, then, why should not the walls unite if they are in condition to have pabulum thrown out? He says because it will be washed away. Suppose that was partially true, would that prevent union?

Cut the gums, they are bathed in the saliva, still we have union. There is not enough secretion to wash away the pabulum, if it all passed through, which it does not, for some of it escapes through the nostril. But if it all passed through it would not be sufficient to wash away the pabulum, and prevent the walls uniting.

*Dr. Rawls.*—That will do with soft tissues but it won't do with bone tissue. The soft parts may come together, and there may be union, but that is not the case with the bone. Many times we think there is union when there is not. It may look as though it was closed, but if you examine closely you will find an opening.

*Dr. H. A. Smith.*—The bone is protected by the soft parts. Would not the flow that would prevents formation of bone prevent the restoration of the living membrane? I would like to ask Dr. Peabody if in his case the opening is closed now.

*Dr. Peabody.*—That is a matter of doubt. I could not introduce a slip of paper. Yet water would come through. Don't know whether it is closed or whether the bone has become so dense that it prevents the passage of foreign matter.

Subject passed.

### THURSDAY.

#### MORNING SESSION.

*Dr. Rawls* —Mr. President I feel somewhat ashamed, not having a paper prepared on this subject. The subject of Therapeutics is one of exceeding interest to all—whether it is or not we generally start in with that remark. But it is, in fact, one of great interest. It is of interest from the fact that we frequently use remedial agents, not knowing just what the effect—the action—of the medicine is. It is true we have many useful remedies, that we know the result, if given, but some of them we do not know how they act We give certain things and they excite and cause extra circulation. Can we tell how they act? Can we tell why? When we give certain remedies to one patient they give great relief, while in other cases they will not relieve, and not only not relieve, but sometimes produce unexpected difficulties. Take the case of counter irritants. To one patient they will give great relief, while in another they will make a scar that will disfigure for life. We can't tell how or why.

Now, there is a reason for this. Is it because of the remedies we apply, or is it that the condition of the circulation is such in the part that it fails to draw the blood away from the diseased part, or do we use it in such strength as to cause abscess? What is the action of iodine? Some say stimulant. Is that all the action of iodine? We know the favorite theory is iodine increases the action of the absorbents. How then does this take place? Antiseptics. We use carbolic acid and salicylic acid as antiseptics. Some say they are not escharotic. Salicylic acid is only a modified form of wood creosote.

You apply salicylic acid to the mucuous membrane and it will coagulate the albumen. Some persons are in the habit of using it for a mouth wash in small quantities. Largely diluted this may do, but to use strong solution would be objectionable. Great physiological and therapeutic effects may be produced by pressure. This may be and is used as a great remedial agent. Pressure will cause absorption. We are in the habit of using it to cause absorption. In correcting irregularities of the teeth why not apply pressure for reducing swelling? In the action of pressure on inflamed surfaces we have the cure for many diseases. There is a good deal to be said on the subject of Therapeutics, and I don't know that I am capable of saying it. But if medical men would dip down deeper, not only they, but the world would be greatly benefitted. They cannot tell why such and such remedies will do certain things. They know it is done, and that satisfies them. When we are able to tell what the action is we will be better able to practice dentistry, and until then we are more or less in the dark.

*Dr. Peabody.*—Mr. President, I would like to ask if any one has ever used chloral hydrate in abscess.

*Dr. Van Antwerp.*—I have tried it. A woman came into my office one day, seemed to be suffering very much from pain, face swollen. She was exceedingly nervous. She was the wife of a physician of our county, and I was anxious to give her relief immediately, if possible, and I selected some chloral hydrate and camphor. I intended to add glycerine, but found it made a very good solution without it, just the consistency I wanted. I took a hyperdermic syringe and injected three drops. I gave her the vial and told her to rub some of the solution on the gum with her finger occasionally. I saw her a few days afterward, swelling gone, no pain. She told me it gave almost instant relief. There is one thing, the relief might have come from opening up the parts, but the after results were more than I expected. And when I removed the cotton and cleansed and filled the root with cotton saturated with the solution, covering it over with cotton saturated with gum sardarac. When I removed the cotton it was perfectly dry. No odor, but camphor, and all doing well. I renewed the application, and the next time she came I filled the tooth with Ox. chlor of zinc.

*Dr. Peabody.*—I did not refer to camphor chloral. I suppose we all understand the action of camphor chloral. But I want the action of chloral hydrate, in solution.

*Dr. Edwards.*—I have used chloral hydrate with very satifactory results internally, in cases of alveolar abscess. When the soft parts were very much swollen, the patient suffering intense pain, very anxious and nervous, and would not permit to even touch the tooth, or adjacent parts. There being nothing else for me to do but administer an anodyne to obtain rest and sleep, have prescribed the chloral hydrate in small doses to be repeated in one or two hours, if necessary, until quiet and sleep was obtained. The following morning my patient was quiet and refreshed, the abscess pointed and ready for the knife, which the patient would submit to willingly. Have never used chloral hydrate locally, though I have used chloroform and camphor in this way.

*Dr. Peabody.*--I have used chloral hydrate internally, frequently, and always with excellent results. Patients suffering great pain, nervous and irritable, give them thirty grains chloral hydrate.

*Dr. Cassidy.*—Is not that rather a large dose, Doctor?

*Dr. Peabody.*—No, sir. Don't think it is. It is given in much larger doses.

*Dr. Rawls.*—No, sir. I like solution about one hundred and twenty grains to the ounce. It has been given as high as 100, 120, 140, and 200 grains. Gov. Morton, of Indiana, was given two hundred grains inside of two hours. So was Chas. Sumner, when he was so ill in Washington, given very near two hundred grains inside of two hours.

*Dr. Peabody.*—I have frequently given twenty grains. Wait for an hour, and if no result, repeat it. I would like to hear from Dr. Sutphin on this subject.

*Dr. Sutphin.*—I often give from ten to fifteen grains, and from that on, as high as forty grains. I wouldn't like to give over forty grains. Of course it might be given in very large doses and have no bad effect. There is a case on record where ten grains proved fatal. Sometimes we have to give larger doses of a great many things, that in an ordinary case we wouldn't think of doing. I once gave a patient seven teaspoonsfull of laudanum before I could relieve her. But that was an extraordinary case. Would not like to give more than forty grains at a dose; it is a very powerful soporific; I would use it very carefully as a dentist. We can't get at these things. We can't tell why calomel acts on the liver. Why buchu acts on the kidneys. Should use chloral very carefully as a dentist.

OPERATIVE DENTISTRY.

*Dr. A. W. Smith.*—I hoped to present a case of osteo-sarcoma before the Association, and am very much disappointed. My patient is not able to be present. In Operative Dentistry there are many things we might speak of. I have no written report, as I intended. I will speak in regard to the preparation of gold. Some of you are familiar with my manner of preparing gold. I cut the foil in strips and then make a rope. I roll it on spunk with the edge of the shears. I am satisfied it is better than pinching up or rolling it with the fingers, for in doing so the crispness is taken out of it, you harden it, and in working, it does not pack closely. I then pass the rope through the flame, and pack in, and with the rope you will have a kind of arched form. They will be firm and not yield under the pressure of the mallet. I then take the electric mallet, and by touching a small spring you will have three thousand taps a minute, perhaps—the gold yields under the pressure. And I will make a perfectly firm and solid filling. You can make a more perfect filling in this way than with any other force. With the electric mallet the force is always the same; with any other it is unequal, and you are apt to injure the structure. The curves must be broke down, and this is effected with the electric mallet.

I wish to speak and commend Dr. Peabody's manner of filling roots with lead. It is now getting to be old practice. I have used it in quite a number of cases, and in cases of abscess it always produced good effect.

*Dr. Rawls.*--I would like to ask Dr. Smith what is the therapeutic action of lead in the roots of teeth.

*Dr. A. W. Smith.*—It is my opinion that the sulphide of lead is formed. I consulted Prof. Tobin about it, and he was of the same opinion. Dr. Peabody thinks that is a mistake. From observation I came to that opinion. Prof. Tobin is still of that opinion. Any way, the general result is good.

*Dr. Peabody.*—I stated to Prof. Diehl, chemist, of our city, what Prof. Tobin had said. He said he thought that almost impossible. Spoke to several, they all thought it impossible. The only cases I have failed in have been inferior teeth. Would like to know what has been the experience of others.

*Dr. Taft.*—I took up lead at the suggestion of Dr. Peabody. Have used it in a number of cases; I do not remember a single failure. Have used it in quite a number of cases of abscess. In all of them there was an improvement, and in some an entire eradication of the disease. My method of employing it is as follows: I open the canal, take the lead wire, cut off a piece about the right length, and roll it with a file. I have always intended to draw some out with draw-plate, but have never done it. Then try it in the canal; see where it rubs; trim it off, and try again, until it fits; then drive it up until it occupies all the space. If there is any better way I would like to know it. This is about the way I generally use it. If you apprehend it will not fill the root, a very good way is to dip the lead in solution of gutta-percha, and drive up well, and you can feel pretty sure it is all filled.

*Dr. Edwards.*--The question, if I understand it correctly, is the therapeutical action of lead. Now, if I understood Dr.

Peabody correctly some two years ago, there can be no therapeutical action at all. He stated a case where he had extracted a tooth, which had been filled for a year or two. He found the lead wire had passed through the foramen about the sixteenth of an inch, and when the tooth was extracted the lead was as bright and the edges as sharp and well defined as the day his knife divided it. This fact shows plainly there had been no action on the lead, therefore there could be no salt of the metal to produce a therapeutical effect. About thirteen years ago I heard Dr. Gorgas lecture on materials suitable for filling root canals, one of which was lead. I think the results of lead as a filling is not so much due to a therapeutical action as to its perfect adaptability to the canal. My mode of preparing it is similar to that described by Dr. Peabody.

*Dr. Taft.*—After the wire, as nearly adapted to the canal as possible, cut the end so it will not quite reach the apex of the root, then cut the wire of the proper length and drive it up. The results in this way are better than by having a long wire, as a long piece is apt to double on itself, and in that way not make as perfect a filling. In this way I am assured that the apical end of the root is thoroughly filled. Particles of other filling materials are more liable to pass through the foramen and produce irritation. Lead is more easily controlled.

*Dr. Peabody.*—I suppose I have had about as much experience with this material as almost any one. When I first introduced the subject I intended it mostly for sluggish abscesses, old chronic cases. Some years ago I used quite small pieces, of later years I have used it as Dr. Edwards suggests. I would rather it would pass through the foramen half a line than not have it go quite up to the apex. There are many materials with which we can make as perfect fillings as lead. We can fill roots with gutta-percha, or any of the plastic fillings, and make as perfect filling, and yet not have the same results as with lead. Some time ago I filled a tooth. I saw the case afterwards, and noticed the tooth was turning black, thought it leaked, told the gentleman to come in and let me examine it. I examined it, found the filling intact. I was perfectly non-plussed, took out the gold, drove up the lead.

Considerable chemical action had gone on there. I put one drop of hydro-chloric acid in the tooth, let it remain about a minute. There was some change at the neck but nowhere else, and very little there, coloring evidently gone through to the enamel. I tried everything I could think of but could not restore it; the tooth is blue to-day. There had evidently been a leak in the root filling, and I think the secretions had seeped down and produced the discoloration.

There are many instances where other fillings passed through the foramen; remember one tooth I extracted, root filled with ox. chloride of zinc; it had passed through the foramen and formed a regular button on the end of the root. I am confident there is something more than adaptation of the lead that causes that change. If that was all, why will not filling of ox. chlor of zinc make such change? I have filled roots where the pus was flowing and worked an entire change.

You misunderstood me about the discoloration of the lead where cut. I said the angles not changed--showing there was no solution of the lead—the foramen accordingly filled and prevented any flow of the secretion.

*Dr. A. W. Smith.*—I think the solution of the matter is found in the therapeutical effect. I think there is a salt formed, the sulphide of lead and there is some therapeutical action. I think the opinion of Dr. Edwards not correct. I have been experimenting in replanting. I had one case. I extracted, a molar tooth. I might have saved it by just the ordinary practice, but I deemed it best to extract. Cleansed out thoroughly and filled the roots with lead, the crown with gold. When I came to replace it I found I could not on account of the palatine roots standing out so far. I cut the palatine root one-third off, and then put the tooth in its place. It is a success. I would like to know how long these teeth generally last. That tooth I treated four years ago. Some member, last year I think, said he had one that had been in five years. How long will they last?

*Dr. Edwards.*—I don't think the case Dr. Smith cited proves any therapeutical action in that case. I saw a case some months ago where a lady had had a tooth filled with amalgam just as healthy as any tooth in the mouth. The tooth had never given her any trouble, but the cavity was in a very difficult place to reach, and the operator suggested it would be a good plan to extract it and fill. Without thinking of the pain of extracting, she said very well, and he took it out, prepared it, and filled with amalgam. It was filled about two years ago, and when I saw it, some three or four months ago, it was doing well, perfectly healthy in every way, gum and everything about it. Nor does the case of Dr. Peabody prove any action of the lead. The blue coloring don't show anything. Who of us have not seen cases where the whole tooth was blue and no lead about it?

*Dr. Taft.*—Now in regard to replanting there is great diversity of opinion. For some time many were sanguine, but after a while they found the roots were gradually dissolving away from some cause. What is the cause of this? No one as yet has ventured to give an opinion. Very little is known as to the cause. Nevertheless the fact occurs in many cases. I had a letter from Dr. Morrison a short time ago who has had about as much experience as any other person. He states quite a number of his cases has undergone this change, and that he fears for even the very best. Some have had great success, while others have not. Prof. Watling, who has had some experience, says he has become quite discouraged as to the final results. This is a very important operation. Can it not be made more sure? Is there not some treatment to be found to secure this, and would it not be well to fill the roots with lead? This result does not occur in all cases. If we can discover some means to make the operation more successful it will be quite an advanced step. I have little doubt we will finally find some means by which to overcome this trouble.

*Dr. A. W. Smith.*—Mr. President, Prof. Tobin is present. I would like to hear from him on the action of lead, as referred to in these discussions.

*Prof. Tobin.*—The reaction of metallic lead upon the organic structure of the tooth may be considered, 1st Physically, 2nd Chemically, and 3rd Physiologically.

*First.* The contact of dissimilar bodies, being conductors of electricity, under certain conditions, generate the electro-motive force and produce an electric current. Ganot defines the essentials of an electric current to be two dissimilar conductors in contact with each other and an exciting fluid. In the law of Ohm the electro-motive force of any such combination is expressed as being directly proportioned to the dissimilarity of these elements and the intensity of the exciting or corroding fluid. Electro-motive force will therefore represent waste or destruction in the elements of the combination.

Now although some of these conditions are present when lead is introduced into the organic structure of a tooth, others are not. We have two bodies, lead and bone, in contact, and we may have an exciting action in the fluids of the mouth or secretions. One of the elements, however, bone, is a non-conductor, hence at least one of the conditions being absent an electric current cannot be established. There being no current there can be no electro-motive force consumed and therefore no waste.

*Second.* The chemical action of the animal fluids upon lead may be of a very complex nature. It may further be modified by the constitution and characteristics of the patient or by abnormal secretions. The salts of lead, likely to be formed, are mostly insoluble or sparingly soluble in water. The soluble salts of lead only are blood poisons. The pollution of drinking water, by passing through lead pipes, has been the subject of considerable attention amongst chemists. Their results may be of use in this connection, and are as follows; "(1) Pure water, free from air, does not act upon metalic lead. (2) If water contains air the lead oxidizes, forming a slightly soluble hydrate. (3) If, in addition, the water contains chlorides, nitrates, or decomposing organic matter, the oxide formed is dissolved and the water may be seriously contaminated with poisonous lead salts. If on the other hand, (4) the water contains phosphates, sulphates, or carbonates, insoluble salts will be formed which will protect the surface of the metal from further chemical action. Excess of carbonic acid will again render the carbonate of lead slightly soluble. (5) Hydrogen sulphide forms an insoluble compound with lead." I

am inclined to think that in a healthy condition of body one or more of the insoluble compounds would be formed, viz., the carbonate, phosphate, or more probably the sulphide, and thus forming a protective surface, render lead free from further corrosive action in the tooth.

*Third.* The physiological effect of any special foreign matter inserted into the animal system is a subject that would better come in the scope of special experiment and observation. Lead bullets has been known to remain years in the flesh without specially affecting the subject. Whether in contact with other secretions different effects might arise, is a matter purely the subject of research, and should be sustained by close and discriminate observation. I would suggest that teeth which have had lead fillings introduced and the teeth afterwards extracted, should be minutely examined physically with the microscope and chemically with the blowpipe or otherwise to ascertain what formation has taken place; at the same time the general condition of the patient as well as the healthy or abnormal condition of local organism: all of which being accurately recorded would form an invaluable basis for the induction of a general theory that would prove invaluable in determining whether in any or all cases lead might be introduced into the teeth with safety.

